# Basis for changes in the disease burden estimates related to vitamin A and zinc deficiencies in the 2017 and 2019 Global Burden of Disease Studies

**DOI:** 10.1017/S1368980021004821

**Published:** 2022-08

**Authors:** Sonja Y Hess, Alexander C McLain, Haley Lescinsky, Kenneth H Brown, Ashkan Afshin, Reed Atkin, Saskia JM Osendarp

**Affiliations:** 1 Institute for Global Nutrition, Department of Nutrition, University of California, Davis, CA 95616, USA; 2 Department of Epidemiology and Biostatistics, Arnold School of Public Health, University of South Carolina, Columbia, SC, USA; 3 Institute for Health Metrics and Evaluation, University of Washington, Seattle, WA, USA; 4 The Micronutrient Forum, Washington, DC, USA

**Keywords:** Global Burden of Disease, GBD Study, Vitamin A, Zinc, Deficiency

## Abstract

**Background:**

The Global Burden of Disease (GBD) Study provides estimates of death and disability from eighty-seven risk factors, including some micronutrient deficiencies.

**Objectives:**

To review methodological changes that led to large differences in the disease burden estimates for vitamin A and Zn deficiencies between the GBD 2017 and 2019 Studies.

**Methods:**

GBD publications were reviewed; additional information was provided by GBD researchers.

**Results:**

Vitamin A deficiency prevalence is based on plasma retinol concentration, whereas the estimate for Zn deficiency prevalence uses dietary adequacy as a proxy. The estimated global prevalence of vitamin A deficiency for children aged 1–4 years in the year 2017 decreased from 0·20 (95 % CI 0·17, 0·24) in GBD 2017 to 0·16 (95 % CI 0·15, 0·19) in GBD 2019, while the global prevalence of Zn deficiency did not change between the two studies (0·09 (95 % CI 0·04, 0·17) and 0·09 (95 % CI 0·03, 0·18)). New to 2019 was that meta-analyses were performed using Meta Regression – Bayesian, Regularized, Trimmed, a method developed for GBD. Due to this and multiple other methodological changes, the estimated number of deaths due to vitamin A deficiency dropped from 233 000 (179 000–294 000) to 24 000 (3000–50 000) from GBD 2017 to 2019, and for Zn deficiency from 29 000 (1000–77 000) to 2800 (700–6500), respectively.

**Conclusion:**

The changes in the estimated disease burdens due to vitamin A and Zn deficiencies in the GBD reports from 2017 to 2019 are due primarily to changes in the analytical methods employed, so may not represent true changes in disease burden. Additional effort is needed to validate these results.

Vitamin A and Zn deficiencies remain public health concerns in low- and middle-income countries, as they are prevalent among vulnerable population groups; according to several published meta-analyses, both micronutrient deficiencies increase the susceptibility to and/or severity of common childhood illnesses, such as diarrhoea and pneumonia^(1–4)^. Efforts are under way to prevent these deficiencies through fortification and supplementation programmes^(5–7)^. It is important to assess both the deficiency prevalence and the related burden of disease to determine the need for intervention programmes and track progress towards disease prevention.

The Global Burden of Disease (GBD) Collaboration led by the Institute for Health Metrics and Evaluation (IHME) has been producing the GBD Study, which estimates death and disability from more than 369 diseases and injuries and from eighty-seven risk factors for 204 countries and territories^(8,9)^. The GBD Collaboration uses two main approaches to estimate the global micronutrient disease burden: (1) micronutrient deficiency as an underlying cause of disease (e.g. blindness due to vitamin A deficiency)^(8)^ and (2) micronutrient deficiency as a risk factor for other diseases (e.g. diarrhoea attributable to vitamin A deficiency)^(9)^. The modelling strategies for these two approaches differ, but in both approaches the first step is to estimate the prevalence of the micronutrient deficiency of interest^(10)^. In the risk factor approach, which is used to estimate the attributable deaths and disability-adjusted life-years, the disease burden and mortality estimates are based on findings of meta-analyses of randomised controlled trials of the effects of providing additional amounts of the MN and are modelled specifically for the population attributable fraction of a target population group that is deemed to be deficient. Both vitamin A and Zn deficiencies are considered as risk factors for morbidity and mortality in young children in the GBD Study.

The GBD Collaboration regularly updates the GBD Study for publication in The Lancet^(11)^, and each GBD Study supersedes previous GBD Studies because of changes in available data, model assumptions and analytical methods used to produce the estimates. In the most recent GBD 2019 Study^(9)^, the estimated GBD due to vitamin A and Zn deficiencies decreased dramatically from the previous GBD 2017 Study due primarily to methodological changes^(12)^. The estimated global prevalence of vitamin A deficiency decreased from the GBD 2017 Study to the GBD 2019 Study both for all ages and for children 1–4 years of age, while the global prevalence of Zn deficiency for children aged 1–4 years did not change from one study to the next (Table 1). The estimated number of deaths due to vitamin A deficiency dropped from 233 thousand (179–294 thousand) in the GBD 2017 Study to 28 thousand (3·1–58 thousand) in the GBD 2019 Study^(12,13)^. Similarly, the estimated number of deaths due to Zn deficiency in 2019 (3·5 thousand (0·9–7·9 thousand) was markedly lower compared with the GBD 2017 Study (29 thousand (1–77 thousand))^(12,13)^. Explanations for these differences are not included in the published reports. Recognising the considerable influence of the GBD Study publications for policymakers and funding agencies, it is important to understand the drivers behind these results. Thus, the present review aims to summarise changes in the methodology used by the GBD Collaboration, which resulted in these differences. We will not perform a comprehensive review of every aspect of the GBD analyses related to micronutrient deficiencies.

**Table 1:** Comparing the estimated global burden associated with vitamin A and Zn deficiencies in the GBD 2017 and 2019 Studies. The measurement year is equal to the GBD Study year unless noted otherwise

	GBD Study 2017^(12)^		GBD Study 2019^(9)^	
	Mean	95 % CI	Mean	95 % CI
Vitamin A deficiency				
Prevalence of vitamin A deficiency (aged 1–4 years)				
2017	0·20	0·17, 0·24	0·16	0·15, 0·19
2019			0·16	0·14, 0·17
Prevalence of vitamin A deficiency (all ages)				
2017	0·11	0·10, 0·12	0·068	0·065, 0·071
2019			0·063	0·061, 0·066
Deaths (thousands) due to vitamin A deficiency	233	179, 294	24	3, 50
YLD (thousands) due to vitamin A deficiency	8810	5768, 12 813	1222	833, 1711
DALY (thousands) due to vitamin A deficiency	28 992	23 040, 35 614	3297	1347, 5594
SEV due to vitamin A deficiency	1·88	1·61, 2·21	15·01	13·55, 16·86
% of total DALY due to vitamin A deficiency	1·16 %	0·95 %, 1·39 %	0·13 %	0·06 %, 0·22 %
Rank of risk due to vitamin A deficiency*				
in DALY among aged 1–4 years only	4		7	
in deaths among aged 1–4 years only	6		7	
in DALY among all ages	16		36	
in deaths among all ages	24		36	
Zn deficiency				
Prevalence of Zn deficiency (aged 1–4 years)				
2017	0·09	0·04, 0·17	0·09	0·03, 0·18
2019			0·09	0·03, 0·18
Deaths (thousands) due to Zn deficiency	29	1, 77	2·8	0·7, 6·5
YLD (thousands) due to Zn deficiency	131	49, 254	17	5, 39
DALY (thousands) due to Zn deficiency	2579	235, 6750	259	67, 597
SEV due to Zn deficiency	0·56	0·17, 1·11	8·78	2·89, 17·60
% of total DALY due to Zn deficiency	0·10 %	0·01 %, 0·28 %	0·01 %	0·003 %, 0·02 %
Rank of risk due to Zn deficiency*				
in DALY among aged 1–4 years only	8		12	
in deaths among aged 1–4 years only	7		10	

Results are shown as mean (95 % CI).

DALY, disability-adjusted life-years; SEV, summary exposure value, YLD, years lived with disability.

* Rank among all level 3 risks. The only difference in the GBD Study 2019 risk ranks for the years 2017 and 2019 was the rank due to vitamin A deficiency in DALY for all ages, which was 33rd for 2017 and 36th for 2019.

## Methods

The present review was performed in 2020 as part of an informal engagement by the Micronutrient Forum and the IHME with the goal to increase understanding and awareness of the GBD estimates for micronutrient deficiencies and to provide technical assistance to IHME. The published GBD 2017 and 2019 Studies were studied in-depth and results available online were accessed^(9,12,13)^. Additional information and input were provided by IHME.

### Comparison of methodology used in the Global Burden of Disease 2017 and 2019 Studies

Serum retinol concentration (< 70 μmol/l) was used to define vitamin A deficiency in both the GBD 2017 and 2019 Studies^(9,12)^. The prevalence of vitamin A deficiency was estimated for all ages and for specific age groups using nationally representative surveys available in the Vitamin and Mineral Nutrition Information System compiled by the WHO and from Demographic Health Surveys, and other data sources^(14,15)^. These data sources were used in conjunction with modelled estimates of the administration of high-dose vitamin A supplementation for the vitamin A deficiency prevalence model^(5,15,16)^. Other changes in the 2019 model were the inclusion of location-level stunting information as a covariate for the vitamin A deficiency prevalence model and updating both the input data and the statistical method for estimating the supplementation coverage and prevalence of deficiency (Table 2). In GBD 2019, spatio-temporal Gaussian process regression was used instead of DisMod-MR 2.1 (GBD’s standard Bayesian meta-regression tool) to better capture time trends in the data. The estimated global prevalence of vitamin A deficiency for all ages decreased from 0·11 (95 % CI 0·10, 0·22) in the GBD 2017 Study to 0·068 (95 % CI 0·065, 0·071) in the GBD 2019 Study (Table 1).

**Table 2:** Overview of methods used for vitamin A and Zn deficiencies

Risk factor	GBD Study	Prevalence estimation	RR input	RR estimation	Linked disease endpoints
Vitamin A deficiency	2017	DisMod-MR 2.1	RR of individual RCT estimated by Imdad *et al*.^(2)^	Metafor package in R^(20)^	Diarrhoea, measles and LRTI
	2019	ST-GPR	RR as published by individual RCT, only used RR from Imdad *et al*.^(2)^, if original RR not available	MR-BRT^(26)^	Diarrhoea and measles
Zn deficiency	2017	ST-GPR	RR of individual RCT estimated by Mayo-Wilson *et al*.^(4)^	Metafor package in R^(20)^	Diarrhoea and LRTI
	2019	ST-GPR	RR as published by individual RCT, only used RR from Mayo-Wilson *et al*.^(4)^, if original RR not available	MR-BRT^(26)^	Diarrhoea

DisMod-MR 2.1, Disease Modelling – Meta-regression; LRTI, lower respiratory tract infection; MR-BRT, Meta Regression – Bayesian, Regularized, Trimmed; RCT, randomised clinical trials; RR, relative risk; ST-GPR, spatio-temporal Gaussian process regression.

Because there is a lack of representative data available on plasma Zn concentration from different populations, the prevalence of Zn deficiency is estimated based on dietary intake data from nationally and sub-nationally representative nutrition surveys and from food availability data obtained from Supply Utilization Accounts prepared by the Food and Agricultural Organization of the United Nations after adjusting for food waste^(17)^. This information is then used to predict the mean Zn intake at the population level and to characterise the distribution of Zn intake, as a proxy for Zn status^(9)^. The prevalence of Zn deficiency among children aged 1–4 years was modelled based on the estimated risk of inadequate dietary Zn intake in both the GBD 2017 and 2019 Studies (Table 2). Both studies estimated the mean Zn intake using a spatio-temporal Gaussian process regression framework with lag-distributed income as a location-year covariate (the GBD 2019 added energy availability), which was then used to characterise the population distribution of intake in preschool children and determine the proportion of the children with intake of less than 2·5 mg Zn per day, which is the estimated average requirement for Zn in this age group^(18)^. Starting with the GBD 2017 Study, the GBD Collaboration no longer accounts for phytase content in foods as the GBD estimates are limited to young children aged 1–4 years, and a recent model by Miller *et al.* using multiple stable-isotope studies of Zn absorption among young children found no detectable effect of phytate on Zn absorption^(19)^. The estimated prevalence of Zn deficiency among children aged 1–4 years was 0·09 (95 % CI 0·04, 0·17) and 0·09 (95 % CI 0·03, 0·18) for the GBD 2017 and GBD 2019, respectively (Table 1).

In the GBD 2017 Study, the relative risks (RR) of selected illnesses due to vitamin A and Zn deficiencies were obtained by pooling the RR from studies included in the most recently published Cochrane meta-analyses^(2,4)^. In these published meta-analyses used for the GBD 2017 Study, the measurements of risk for each trial were calculated by Imdad *et al.*^(2)^ for vitamin A and Mayo-Wilson *et al.*^(4)^ for Zn, and the GBD Collaboration performed the meta-analyses with the metafor package in R^(20)^. In contrast, the GBD 2019 Study used the reported RR of the individual randomised controlled trials, if available (Table 3). Further, the GBD 2019 Study included additional studies when available^(21–25)^. For the GBD 2019 Study, the meta-analyses were completed using Meta Regression – Bayesian, Regularized, Trimmed (MR-BRT), a method developed by IHME^(26)^. The main feature that sets MR-BRT apart from other meta-analysis approaches is the use of ‘trimmed’ criteria which ignore certain outlier data points (i.e. studies). In general, MR-BRT has a parameter that specifies how much ‘trimming’ is done (i.e. how many data points are ignored). Once this parameter is set, the model and data points to be ignored are estimated simultaneously. For GBD 2019, MR-BRT was applied with 10 % trimming of the data, which had minimal effects on the results.

**Table 3:**
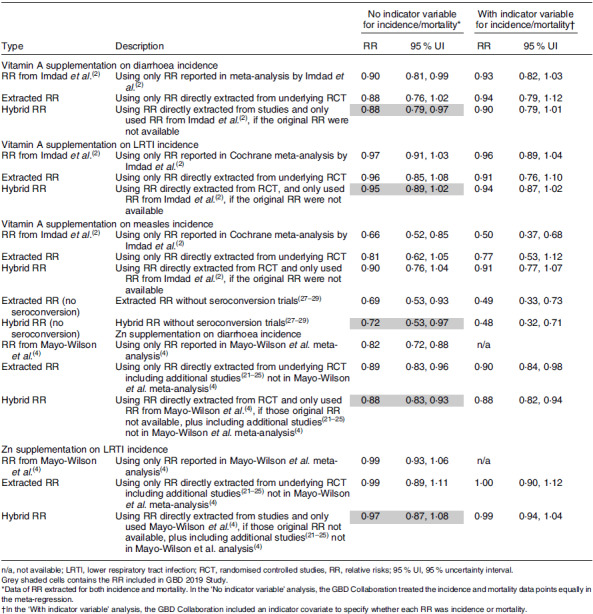
Sensitivity analyses of the relative risks of vitamin A and Zn supplementation on the incidence of diarrhoea, lower respiratory tract infection and measles in the GBD 2019 Study

The methodological updates for GBD 2019 resulted in a decrease in the estimated RR for morbidity *v*. those from GBD 2017. Notably, the GBD 2019 removed lower respiratory tract infections (LRTI) as an outcome of vitamin A and Zn deficiencies because the RR was no longer statistically significant. Further, the RR for diarrhoea was lowered to 1·14 (95 % CI 1·03, 1·26) from 2·35 (95 % CI 2·17, 2·54) for vitamin A and to 1·14 (95 % CI 1·07, 1·21) from 1·90 (95 % CI 1·52, 2·33) for Zn. Measles was included as an outcome for vitamin A in both GBD Studies, but the RR was lowered to 1·39 (95 % CI 1·03, 1·90) in GBD 2019 from 2·76 (95 % CI 2·01, 3·78) in GBD 2017. This is partly due to the exclusion of three studies which only had seroconversion as primary outcome instead of measles-related morbidity or mortality^(27–29)^. For vitamin A and Zn, the GBD 2017 Study adjusted for a relationship between the prevalence of deficiency and the size of the RR. This adjustment resulted in larger RR from studies in locations with higher deficiency prevalence, leading to a larger pooled RR. In the GBD 2019, this adjustment was removed due to finding no statistically significant relationship between the background prevalence of deficiency and morbidity outcomes. Of all the methodological changes, this latter change had the largest impact on the final RR included in GBD 2019 Study.

## Discussion

We have reviewed data and methods used in the two most recently published GBD Studies to provide insight into why the estimated deaths and disability-adjusted life-years due to vitamin A and Zn deficiencies were reported to be substantially lower in the GBD 2019 Study compared with the GBD 2017 Study^(9,12,13)^. For vitamin A, it appears that the reason for the reduction in the estimated disease burden from the GBD 2017 to the 2019 Study is a combination of decreased deficiency prevalence estimates and reductions in the RR of selected diseases. For Zn, it is clear the reduced estimated burden is due just to changes in the RR used in the GBD Study as the prevalence estimates were similar in both the GBD 2017 and 2019 Studies, albeit possibly underestimates of the true deficiency prevalence, as described below. In 2017, the GBD Collaboration relied on the RR published in Cochrane meta-analyses^(2,4)^ and then performed the meta-analyses with the metafor package in R. In 2019, the GBD Collaboration completed their own systematic literature review and meta-analyses with MR-BRT, which were then used in the GBD risk factor model. These MR-BRT meta-analyses used in the GBD 2019 Study have not yet been published, and the description provided in the appendices of the GBD Study published in The Lancet^(9)^ did not provide information about the studies that were trimmed (10 % of the studies were trimmed). Thus, the purpose of the present study is to provide more information on the methodological changes and comment on their likely validity.

The use of serum retinol concentration to determine the prevalence of vitamin A deficiency in both of these studies is appropriate, as it is the recommended indicator to assess vitamin A status^(30,31)^. The decrease in the modelled vitamin A deficiency prevalence is not due to changes in serum retinol. Rather the changes in the modelling technique are primarily responsible for the different prevalence estimates. Which of these GBD studies provides the most accurate estimate of the true prevalence of vitamin A deficiency is uncertain.

The prevalence of Zn deficiency in the GBD Study is based on estimates of dietary Zn intake, which are derived primarily from national food availability data. However, plasma or serum Zn concentration is the recommended indicator for assessing population Zn status^(32,33)^. A review of twenty national surveys in low- and middle-income countries with information on both plasma or serum Zn concentration and Zn availability in the food supply found that estimates of percentage population with inadequate dietary Zn availability underestimate the risk of Zn deficiency^(34)^. Thus, the prevalence of Zn deficiency in the GBD Study is likely an underestimation of the true Zn deficiency prevalence^(35)^. However, there are presently only twenty-eight countries with nationally representative data of Zn status among preschool children and of those only seven countries completed more than one survey^(14)^. Thus, to improve estimates of the prevalence of Zn deficiency and the global disease burden due to Zn deficiency, more plasma or serum Zn data from nationally representative surveys are needed.

The reported RR of morbidity related to vitamin A and Zn deficiency are derived from randomised controlled trials in which the impact of vitamin A and Zn supplementation on diarrhoea and LRTI is determined. Possible errors may be introduced by the trimming of studies with the MR-BRT 2019 models, as described above. In addition, the modelling techniques seem to ignore issues of study quality and other possible modifying effects of the response to supplementation. For example, a published meta-analysis of Zn supplementation and LRTI found that the apparent effect of supplementation varies according to rigor of LRTI diagnosis used in the primary studies. In sub-group analyses, the RR for those studies that diagnosed LRTI based on counting respiratory rate or a physician’s examination was 21 % less in the Zn group than in the comparison group (RR, 0·79; 95 % CI 0·67, 0·94; *P* = 0·013, random-effects model)^(3)^. In contrast, the studies that based the diagnosis only on reported rapid breathing or difficulty breathing (without a physician’s examination) found no significant difference between the Zn supplementation and the comparison group^(3)^. Failure to adjust for study quality would likely dilute the apparent effect of supplementation. In addition, recent meta-analyses found that Zn supplementation did not have a significant impact on all-cause mortality^(4)^, but meta-analyses may miss the age-related impact found in pooled analyses. Specifically, when data of three large trials conducted in Tanzania, Nepal and India^(22–24,36,37)^ were analysed by age group (< 12 months or ≥ 12 months), there was a significant 18 % reduction in deaths among Zn-supplemented children older than 12 months of age, but not in the younger children^(3)^.

For the GBD 2019 Study, the GBD Collaboration conducted eighty-one systematic reviews and meta-regressions^(9)^, including the two micronutrients of interest in the present paper. The details of these reviews and meta-analyses, although presented publicly at conferences, are not yet published in peer-reviewed journals and are not fully described in the appendices to the GBD reports. While the GBD Study is fully compliant with the Guidelines for Accurate and Transparent Health Estimates Reporting^(38)^, a common concern raised by critics of the GBD Study is the lack of transparency of GBD imputation methods^(39)^. Providing more details on assumptions and models and publishing models and findings of new meta-analyses in peer-reviewed journals would help increase transparency.

The lower estimated disease burden due to vitamin A and Zn deficiencies due to modelling changes in the latest GBD Study may lead policymakers and funding agencies to conclude that these micronutrient deficiencies are no longer of public health concern. This could result in reduced investments in the prevention of these deficiencies and ultimately undermine the health of population groups at-risk of deficiency. Thus, additional effort is needed to validate these results. Moreover, the use of proxy indicators may obscure the well-recognised issue of data scarcity concerning population micronutrient status^(40)^, and thus discourage investments in much needed micronutrient status biomarker information collected from representative samples of various population groups^(10)^.

## Conclusions

The estimated disease burdens due to vitamin A and Zn deficiencies are significantly lower in the most recent GBD 2019 Study compared with previous GBD Studies. These differences are due primarily to changes in the methods and models used to create these estimates. Whether these differences are real or simply artifacts of the methodological changes remains uncertain and highlights the fact that the GBD Study is continuously evolving and the methods have not been validated with empirical data. One overarching problem is the scarcity of reliable information on the prevalence of key micronutrient deficiencies, based on direct indicators of population micronutrient status at the national, regional and global level. Thus, there is a need to generate this information, using recommended biochemical indicators of micronutrient status collected in nationally representative surveys^(41)^. Only when such information gaps are filled will national, regional and global estimates of the health and mortality burden due to micronutrient deficiencies be more reliable and accurate.
